# Characterisation of a high-frequency gene encoding a strongly antigenic cystatin-like protein from *Trichinella spiralis* at its early invasion stage

**DOI:** 10.1186/s13071-015-0689-5

**Published:** 2015-02-05

**Authors:** Bin Tang, Mingyuan Liu, Libo Wang, Shenye Yu, Haining Shi, Pascal Boireau, Vasile Cozma, Xiuping Wu, Xiaolei Liu

**Affiliations:** National Institute of Parasitic Diseases, Chinese Center for Disease Control and Prevention, Shanghai, Key Laboratory for Zoonoses Research, Ministry of Education, Institute of Zoonoses, Jilin University, Changchun, P. R. China; Jiangsu Co-innovation Center for Prevention and Control of Important Animal Infectious Diseases and Zoonoses, Yangzhou, 225009 P. R. China; Yunnan Institute of Parasitic Diseases, 6 Xiyuan Road, Puer, Yunnan P. R. China; Immunology Laboratory, Pediatric Gastroenterology Unit, Massachusetts General Hospital East, Charlestown, MA 02129 USA; ANSES, Laboratory for Animal Health, Maisons Alfort, France; University of Agricultural Science and Veterinary Medicine, Cluj-Napoca, Romania

**Keywords:** *Trichinella spiralis*, Intestinal infective larvae, Diagnosis, Cystatin

## Abstract

**Background:**

The intestinal phase is the early invasion stage of *Trichinella spiralis* (*T. spiralis*), in which muscle larvae invade intestine epithelial cells and then develop into adult worms to breed newborn larvae. Thus, intestinal infective larvae are first exposed to the immune system of the host, and antigens from the worms may be the earliest marker in the diagnosis of trichinellosis and may contribute to vaccine development to prevent *Trichinella* infections in pigs.

**Methods:**

A cDNA library of intestinal infective larvae of *T. spiralis* at 6 hours post infection (p.i.) was constructed and immunoscreened using serum collected from pigs that were infected with *T. spiralis* at 26 days p.i. *T. spiralis* cystatin-like protein (Ts-CLP) gene encoding a 45.9 kDa protein was cloned and expressed in *Escherichia coli*. The rabbit antisera were generated and used to determine the location of Ts-CLP in the parasite. Transcription levels of Ts-CLP in different developmental stages of *T. spiralis* were observed by RT-PCR. The potential application of recombinant Ts-CLP in diagnosis against *T. spiralis* infection was tested by ELISA. The immune protection of recombinant Ts-CLP protein against *T. spiralis* infection was evaluated in mice.

**Results:**

Thirty-three positive clones were selected from cDNA library, among which 20 clones encoded the same novel cystatin-like protein (Ts-CLP). Immunolocalisation and real-time quantitative PCR revealed that native Ts-CLP was localised primarily to β-stichocytes and that the *Ts-clp* gene was transcribed and expressed in all developmental stages of *T. spiralis*. The recombinant protein rTs-CLP was recognised by pig antiserum as early as 15 days p.i., and could induce protective immunity in mice, with a 61.21% reduction in the number of muscle larvae.

**Conclusions:**

These data preliminarily suggested that Ts-CLP may play an important role in the early infection of *T. spiralis* and that recombinant Ts-CLP protein is a candidate antigen for diagnosis and vaccine development in *Trichinella* infections.

## Background

Trichinellosis is one of the most important food-borne parasitic zoonosis transmitted to humans through the consumption of raw or undercooked meat contaminated with nematodes of the *Trichinella* spp. As one of the most widespread parasites [1], *Trichinella* spp., which can infect many vertebrates, not only lead to enormous economic losses in the animal husbandry and meat industry but also pose a severe threat to public health. It is estimated that millions of people are chronically infected with muscle larvae (ML) that generate ongoing muscular pain [2]. Therefore, meat inspection for *Trichinella* is mandatory in many countries. The cost of inspection of *Trichinella* spp*.* ranges from $0.12 to $2.5 [3] or to $3.0 [4]. In a small slaughterhouse the cost of inspection may reach $10–$15 per pig [5]. According to the report from the National Bureau of Statistics of China in 2012, 697.9 million pigs were slaughtered in China (http://www.stats.gov.cn/tjsj/ndsj/2013/indexeh.htm).

After the ingestion with contaminated meat, infective ML of *Trichinella spiralis* (*T. spiralis*) are released into the stomach of the host. The ML invade the intestinal columnar epithelial cells and undergo four moults to reach sexual maturity. The adult worms (Ad) mate within the intestinal epithelium, and embryogenesis takes place in 3–4 days. The newborn larvae (NBL) are released as early as 4 days p.i. [6], penetrate the intestine and migrate via the blood and lymphatics to skeletal muscle. The NBL then develop into ML, and the infected muscle cells develop into nurse cells that contain a collagen capsule.

Successful protein-based diagnostic methods require antigens that are not only specific but are also produced economically in large quantities [7]. Although excretory-secretory (ES) antigens, a mixture of many parasite-derived products, are immunogenic and immunoreactive, their standardised large-scale production remains difficult. Moreover, ES antigens from ML of *T. spiralis* have a critical weakness- the ‘blind window’ in which anti-*Trichinella* antibodies cannot be detected until 3–4 weeks p.i. [8,9]. Therefore, ELISA and other serological methods cannot replace artificial digestion methods for *Trichinella* detection in slaughtered pigs.

Previous studies have demonstrated that *T. spiralis* express a variety of diverse antigens at different developmental stages [10], and this characteristic may be the main reason why the ES antigens of ML are not recognised by antibodies induced by the parasites during the intestinal phase. Although antigens from *T. spiralis* in the intestinal phase may fill the ‘blind window’, the large-scale production of these natural antigens is not possible because the life cycle of *T. spiralis* cannot be completed *in vitro*.

The identification of stage-specific antigens is a clear requirement in the search for potential antigens for the diagnosis of and immunoprotection against *Trichinella* infections, as well as to obtain a better understanding of the invasion and evasion mechanism of the parasite. Numerous attempts have resulted in the identification of a series of antigens from ML (53-kDa antigen [11,12], 43-kDa glycoprotein [13], 45-kDa protein [14,15], TspSP-1 [16,17], Ts23-2 [18], Serine proteinase inhibitor [19], P49 protein[20]), Ad (20 AD3 and 30 AD3 [21]) and NBL (glutamic acid-rich protein [22]). However, it is noteworthy that none of the reported antigens were derived from intestinal infective larvae which represent the earliest exposure to the host immune system.

In the present study, a high-frequency gene encoding a strongly antigenic cystatin-like protein from *T. spiralis* (*Ts-clp*) during its early stage of invasion (intestinal infective larvae in the intestine at 6 hours p.i. [L6h]), was identified, and its potential applications in diagnosis of and immunoprotection against *T. spiralis* infections were identified.

## Methods

### Animals

BALB/c mice (female, 6–8 weeks old) were purchased from Shanghai SLAC Company. Female Wistar rats, New Zealand white rabbits and Chinese Changbai pigs were purchased from the animal facilities of Jilin University, China.

### Ethics statement

Animals were treated in strict accordance to the National Institutes of Health guidelines (publication no. 85–23, revised 1996). Animals were reviewed and approved by the Ethical Committee of Jilin University affiliated to the Provincial Animal Health Committee, Jilin Province, China (Ethical Clearance number IZ-2009-08).

### Preparation of parasites and ES products

Muscle larvae of *T. spiralis* (ISS 534) were recovered from BALB/c mice at 35 days p.i. Wistar rats were divided into 13 groups with 12 animals per group and infected per os with 10 000 ML. The intestinal infective larvae were isolated from the small intestines of infected rats at 10 min, 20 min, 30 min, 1 h, 2 h, 3 h, 4 h, 5 h, 6 h, 7 h, 8 h, 9 h and 10 h p.i.

The intestinal infective larvae at 24 h p.i. (L24h), adult worms at day 2 (Ad2), 3 (Ad3) and 5 (Ad5) p.i. and NBL were recovered as previously described [10,23]. The ES products of ML, L6h, Ad5 and NBL were prepared according to the method of Liu *et al.* [6]. All of the parasites were washed 3 times in phosphate-buffered saline and stored at −80°C for further use.

### Construction and immunoscreening of an L6h cDNA library

The mRNA was isolated from the total RNA of L6h using the Oligotex mRNA Kit (Qiagen, Germany) and reverse-transcribed into cDNA using ZAP-cDNA Synthesis Kit (Stratagene, USA). After the addition of an *Eco*RI adaptor and digestion with *Xho*I, the digested cDNA was separated using CHROMA SPIN-400 (Clontech, USA), ligated into the λZAP II vector and packaged in Gigapack III Gold packaging extracts (Stratagene, USA). The size and recombination rate of the primary library and amplified library were calculated separately. A total of 10^5^ plaque forming units (pfu) from the amplified library were screened against absorbed antiserum obtained from *T. spiralis-*infected pigs at 26 days p.i. according to Wu *et al*. [24]. Positive clones were selected, and pBlue-Script plasmids containing the cDNA inserts were excised from the λZAP II phage vector. The double-stranded DNA was sequenced from both strands, and the nucleotide and deduced amino acid sequence were analysed using DNA Star software.

### Expression and purification of recombinant protein (rTs-CLP)

The sequence of *Ts-clp* (GenBank: EU263325.1) without the N-terminal signal peptide was amplified from the cDNA of L6h by PCR with primers (forward, 5′-*CCG AAT TCC AGA TAC TTG GTG A*-3′, containing the *Eco*RI restriction site; reverse, 5′-*TAG CTC GAG TTA ACG GAA AAA AGT GA*-3′, containing the *Xho*I restriction site). The PCR products were subcloned into the expression vector pET-28a with *Eco*RI and *Xho*I sites. After transformation into *Escherichia coli* BL21 (DE3) cells (Novagen, Germany), the expression of rTs-CLP was induced with 1 mM IPTG for 6 h at 37°C. The bacterial culture pellet was resuspended in solution (20 mM Tris–HCl, pH 7.5, 10 mM EDTA, 1% Triton X-100). Lysozyme was added to the solution and the mixture was incubated at 37°C for 15 min and then sonicated on ice. Following centrifugation at 3 000 rpm, the soluble and insoluble fractions were obtained and analysed by 12% SDS-PAGE. The proteins were purified using HiTrap™ affinity columns (GE healthcare, USA) according to the manufacturer’s instructions.

### Generation of rabbit antisera

Three New Zealand white rabbits were first injected intradermally with approximately 0.4 mg of the purified rTs-CLP mixed 1:1 (v/v) with CFA. Three additional injections of 0.4 mg of protein with IFA were administered to the animals at one week intervals. The last boosting injection was administered 4 weeks after the first immunisation. Preimmune serum was collected prior to the immunisations. One week after the last injection, serum samples containing the anti-rTs-CLP antibodies were collected, titrated and stored at −20°C.

### SDS-PAGE and Western blot analysis

Equal amounts of samples (20 μg per well) including the ES products of ML, L6h, Ad5, NBL and purified rTs-CLP were loaded onto a 12% polyacrylamide gel. Separated proteins were transferred to Hybond-C extra pure nitrocellulose membranes (Amersham Biosciences, USA). The membranes were blocked with TBST (20 mM Tris · HCl, 130 mM NaCl, pH 7.6, 0.1% Tween-20) plus 3.5% (w/v) BSA at 4°C overnight. The membrane carrying rTs-CLP was cut into strips and incubated with sera (1:400 dilution in TBST) from *T. spiralis*-infected pigs and mice for 1 h at room temperature. Preimmune pig serum served as a negative control, and the serum of rTs-CLP-immunised rabbits was used as a positive control. The membrane carrying ES products was incubated with the serum of rTs-CLP immunised rabbits for 1 h. All of the membranes were washed with TBST and then incubated with the corresponding alkaline phosphatase (AP)-conjugated secondary antibodies (Sigma, USA) for 1 h. Finally, the bands were detected using NBT/BCIP (Promega, USA).

### Immunolocalisation

Intestinal tissue at 6 h p.i., muscle tissue at different days p.i. and worms of Ad3 and Ad5 were prepared [10,23] and washed in PBS. Next, the samples were fixed with 4% polyoxymethylene for 6 h. The paraffin-embedded sections mounted on poly-L-lysine-coated glass slides were deparaffinised for 30 min in xylene, and rehydrated using graded alcohol baths. The slides were treated with 0.1% Triton X-100 for 1 h and then with protease K (20 μg/mL) for 5 min, followed by 3 washes with PBS. The sections were then blocked with 5% sheep serum for 30 min and incubated with a 1:256 dilution of rabbit anti-rTs-CLP serum overnight at 4°C. Next, the sections were washed 3 times in PBS for 15 min with gentle agitation. The sections were incubated with Alexa Fluor 555 goat anti-rabbit IgG for 1 h at room temperature in the dark and then washed 3 times with PBS. Finally, they were incubated with 1 μg/mL Hoechst 33342 for 5 minutes at room temperature to stain the nuclei, and mounted with glycerol. Laser scanning confocal microscopy (Fluoview FV 1000, Japan) was used to examine the sections. The preimmune sera of rabbits served as a negative control.

### Real-time quantitative PCR

Total RNA from ML, L6h, L24h, Ad2, Ad3, Ad5, and NBL were prepared using TRIzol Reagent (Invitrogen, USA). To eliminate genomic DNA contamination, total RNA was treated with RNase-free DNase I (Takara, Japan). The quality and quantity of the RNA were assessed by denaturing 1% agarose gel electrophoresis. The total RNA of ML, L6h, L24h, Ad2, Ad3, Ad5, and NBL was separately reverse-transcribed using M-MLV Reverse Transcriptase (Promega, USA). The glyceraldehyde-3-phosphate dehydrogenase gene of *T. spiralis* (Ts-GAPDH, GenBank: AF452239) was selected as the endogenous control. Real-time PCR was conducted using an ABI Prism 7500 (Applied Biosystems, USA) with Power SYBR green PCR Master Mix (Applied Biosystems, USA) and the following primers: *Ts-clp* forward, 5′-*TAC CCA TCG CAA TCC*-3′; *Ts-clp* reverse, 5′-*CTG CCC TTC CAC CTA*-3′; Ts-GAPDH forward, 5′-*GTG CTG ATT ACG CTG TTG*-3′; Ts-GAPDH reverse, 5′-*CTA AGC CAT TGG TAG TGC*-3′. The threshold cycle (*C*_T_) values were generated with Sequence Detection Software, version 1.3.1 (Applied Biosystems, USA). Three separate real-time PCR assays were performed, and each reaction was performed in triplicate. The transcript abundance of *Ts-clp* was calculated relative to Ts-GAPDH according to the method reported by Pfaffl [25].

### ELISA test

Three Changbai pigs were infected per os with 20000 muscle larvae of *T. spiralis*. The pig sera were collected prior to infection and at 5, 10, 15, 20, 25, 30, 40, 50 and 60 days p.i. The serum samples obtained prior to infection served as the negative controls.

The rTs-CLP and ES products of ML were used to detect the levels of IgG antibodies in the sera of infected pigs by indirect ELISA. Briefly, optimised volumes of ES products (0.5 μg) and rTs-CLP (0.1 μg) in 100 μL of carbonate-bicarbonate buffer (pH 9.6) were added to 96-well ELISA plates (Costar, USA) and incubated overnight at 4°C. The plates were then washed three times with PBST (137 mM NaCl, 17 mM NaH_2_PO_4_, 58 mM Na_2_HPO_4_, 0.05% Tween-20, pH 7.4), blocked with PBST containing 1% BSA for 1 h, and incubated for 2 h at 37°C with 100 μL of a 1:200 dilution of pig serum (in PBST containing 1% BSA). The plates were then washed three times with PBST and incubated with HRP-conjugated goat anti-pig IgG (Sigma, USA). After three washes in PBST, 100 μL of o-phenylendiamine (OPD; Sigma, USA) at a concentration of 0.04% in phosphate-citrate buffer, pH 5.0, containing 0.001% H_2_O_2_ was added to each well. The reaction was stopped after 20 min with 2 M H_2_SO_4_, and the optical density (OD) at 490 nm was measured with an ELISA reader (Biotek, USA). The cut-off value was calculated on the basis of the average OD of negative serum samples plus three standard deviations.

### Immunoprotection analysis

BALB/c mice were divided into three groups of 24 animals each. The first group (immunisation group) of mice was injected intraperitoneally with recombinant protein (20 μg/mouse) emulsified in CFA and boosted with recombinant protein (20 μg/mouse) with IFA at one-week intervals. The second group (adjuvant control group) and third group (PBS control group) were inoculated with CFA mixed with physiological saline or with physiological saline alone. One week after the final booster immunisation, the mice in all three groups were challenged per os with 200 ML of *T. spiralis*. The reduction rates of Ad5 were evaluated by counting the number of Ad5 recovered from 12 mice in each group that were sacrificed at 5 days p.i., while the reduction rates of NBL were assessed by counting the NBL production numbers of female Ad5 (60/group) after *in vitro* cultivation at 37°C for 24 h. The numbers of ML recovered from the other 12 mice in each group sacrificed at 35 days p.i. were used to evaluate the reduction rates of ML. The following formula was employed: reduction rate = (number of worms in the PBS control group –number of worms in the immunisation group)/number of worms in the PBS control group.

### Statistical analysis

Data are expressed as the mean ± SD. Differences between groups were compared using One-Way ANOVA in SPSS 13.0 software (SPSS Inc.), and *P* < 0.05 was considered statistically significant.

## Results

### Optimal time to recover intestinal infective larvae

**Figure 1:**
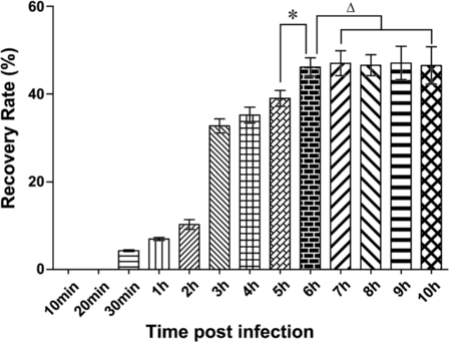
**Determination of the optimal time to collect intestinal infective larvae.** One hundred and fifty-six Wistar rats were divided into thirteen groups and each rat was infected per os with 10 000 ML. The intestinal infective larvae were collected from fractions of small intestines at various time points. ^*^Data are significantly different from the other groups (*P* < 0.05). ^△^Data are not significantly different from groups (*P* > 0.05).

As shown in Figure 1, at 10 min and 20 min p.i., the rate approached zero, and from 30 min to 6 h p.i., the rate increased rapidly from 4.31% to 46.18%. Each group was significantly different compared to the others (*P* < 0.05). The values became relatively stable from 6 h to 10 h p.i. (*P* > 0.05). L6h which invade the intestinal epithelium is a group of intestinal infective larvae from 0 h p.i. to 6 h p.i. and the ES products of these worms were released and exposure to the host immune system. So we preliminarily consider that the L6h is only representative of the larvae at the early stage of invasion, and 6 h p.i. was considered as the optimal time point to recover intestinal infective larvae from the small intestine.

### Molecular characterisation of Ts-CLP

**Figure 2:**
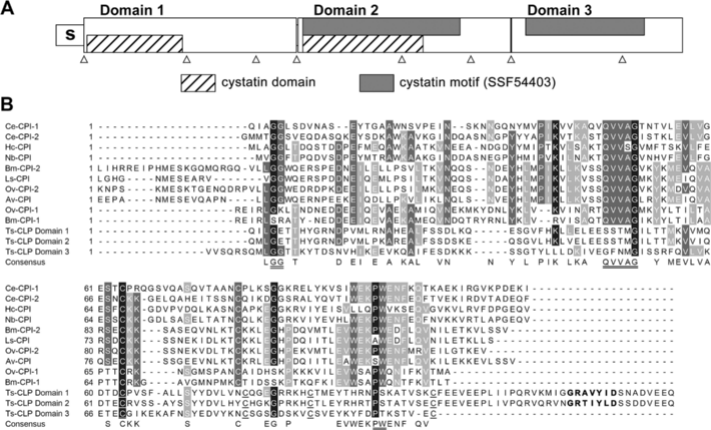
**Bioinformatics analysis of Ts-clp. (A)** Protein sequence analysis of Ts-CLP. Location of each region: signal peptide (s): 1–18; domain 1: 19–156; domain 2: 158–295; domain 3: 296–406; predicted cystatin motif (SSF54403): 161–262 and 305–381. The intron positions within *Ts-clp* are indicated by triangles. **(B)** Alignment of nematode cystatins and cystatin-like proteins. The predicted signal peptide of each sequence was excluded. Evolutionarily conserved sites are double underlined. The four cysteine residues in each domain of Ts-CLP are underlined. Potential auto-processing sites similar to GXNXFXD motif are shown in bold. Accession numbers for cDNA or proteins sequences are as follows: *T. spiralis* - Ts-CLP*,* ABY59464; *Brugia malayi -* Bm-CPI-1, U80972; Bm-CPI-2, AF015263; *Onchocerca volvulus* - Ov-CPI-1, AF177194; Ov-CPI-2, P22085; *Litomosoides sigmodontis*- Ls-CPI, AF229173; *Acanthocheilonema viteae* - Av-CPI, L43053; *Nippostrongylus brasiliensis* - Nb-CPI, AB050883; *Haemonchus contortus* - Hc-CPI AF035945; *Caenorhabditis elegans* -Ce-CPI-1, AF100663; Ce-CPI-2, AF068718.

The size of the primary cDNA expression library of L6h was 1.5 × 10^6^ with a 95% recombination rate, and the titre of the amplified library was 1.2 × 10^12^ pfu/mL. Consequently, the cDNA library was successfully constructed. After immunoscreening with 26-day p.i. pig antiserum, 33 positive clones were selected from 1.0 × 10^5^ plaques, among which 20 positive clones were the same high-frequency cDNA. The clone with longest cDNA insert, contained 1 352 bp with a complete open reading frame (ORF) of 1 218 bp encoding 406 amino acids with a calculated molecular weight of 45,900 and an isoelectric point of 5.43. Ts-CLP consisted of three repeat domains (Figure 2A). Two domains (domain 1 and 2) consisting of 138 amino acids in the N-terminus had an identity of 74%. In addition, the C-terminal domain (domain 3) showed ~37% identity to each of the two domains in the N-terminus. The same cystatin domain was found in domains 1 and 2 by Superfamily (http://supfam.org/SUPERFAMILY), while the same cystatin motif (SSF54403) was detected in domains 2 and 3 by InterPro Scan (http://www.ebi.ac.uk/interpro/).

As shown in Figure 2B, all three domains are different from the cystatins of other nematodes. The highly conserved QXVXG motif of typical cystatins was absent in all three domains, and the PW motif was replaced by a PS in domains 1 and 2 and a PT in domain 3. Four conserved cysteine residues were present in the C-terminus of each domain, by which two disulphide bonds could be formed similarly to the structure of human cystatin C. The signal peptide sequence and N-glycosylation sites indicated that putative peptide might be a secreted glycoprotein.

### Western blot analysis

The molecular mass corresponded well to the predicted size of the gene product. The expression of rTs-CLP was induced in the presence of 1 mM IPTG for 4 hours. The proteins were expressed in inclusion bodies rather than in the supernatant of the induced cells.

**Figure 3:**
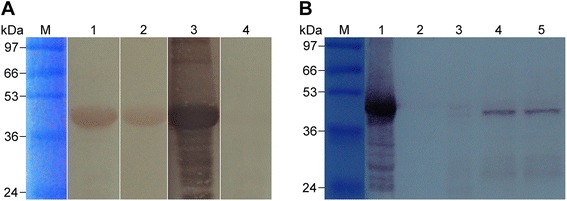
**Western blot analysis of Ts-CLP and its recombinant protein. (A)** The antigenicity of rTs-CLP by Western blot analysis. M: protein standard; 1: sera of mice at 35 days p.i.; 2: sera of pigs at 26 days p.i.; 3: sera of rabbits immunised against rTs-CLP; 4: preimmune sera of pigs. **(B)** Western blot analysis of ES products using the sera of rTs-CLP immunised rabbits. M: protein standard; 1: purified rTs-CLP; 2–5: ES products of NBL, Ad5, ML, L6h.

The purified rTs-CLP was recognised by the sera of *T. spiralis*-infected pigs at 26 days p.i. and mice at 35 days p.i., as well as by antibody against rTs-CLP (Figure 3A). No binding was detected with preimmune pig sera. As shown in Figure 3B, antibody against rTs-CLP in immunised rabbit serum bound to one main band at ~46 kDa and some low-molecular-weight bands in the purified rTs-CLP and ES products of Ad5, ML and L6h. The signal in NBL was not observed.

### Immunolocalisation

**Figure 4:**
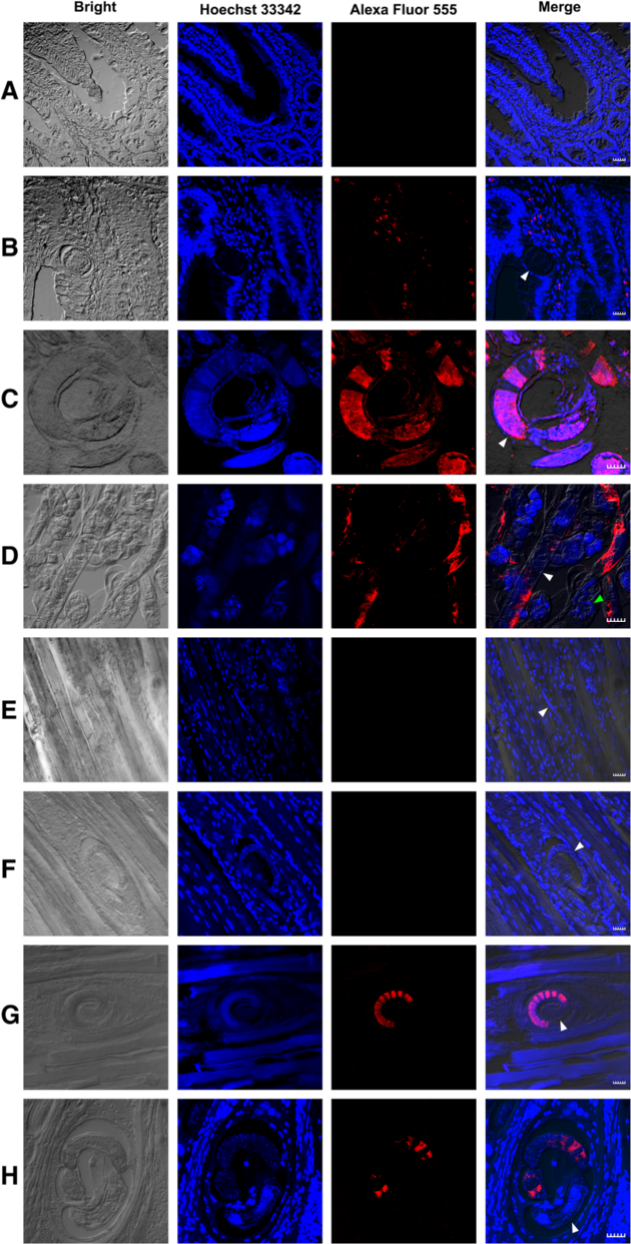
**Localisation of Ts-CLP in paraffin-embedded sections.** Eight micrometre sections were treated with rabbit anti-rTs-CLP serum and Alexa Fluor 555 goat anti-rabbit IgG, then observed by confocal laser scanning microscopy. **A**: normal intestine; **B**: intestine epithelial tissue at 6 h p.i.; **C**: Ad3; **D**: Ad5; **E**: larvae in muscle at 9 days p.i.; **F**: larvae in muscle at 15 days p.i.; **G**: ML at 24 days p.i.; **H**: ML at 60 days p.i. The Ts-CLP signal was red. Bars = 20 μm. The white arrows indicate the worms of *T. spiralis.* The green arrows indicate the NBL of *T. spiralis.*

As shown in Figure 4, signal was detected in specific structures of the worms by confocal laser scanning microscopy. The positive signal in ML and Ad was stronger than that in NBL, whereas it increased by degrees as NBL developed into ML in muscle. At 24 days p.i. signals were clearly located in β-stichocytes (Figure 4G), and remained strong until 60 days p.i. (Figure 4H). It is noteworthy that Ts-CLP signals were observed in intestinal epithelial cells at 6 h p.i. (Figure 4B).

### Real-time PCR analysis

**Figure 5:**
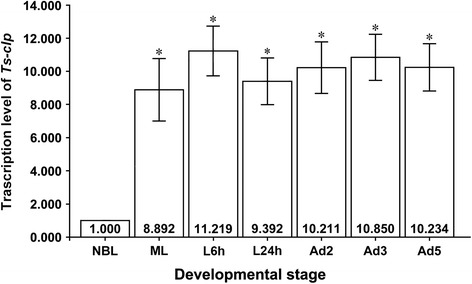
**Real-time quantitative PCR analysis of Ts-clp.** After reverse transcription, the cDNA of ML, L6h, L24h, Ad2, Ad3, Ad5, and NBL was used as a template for real-time quantitative PCR using SYBR Green. Each reaction was performed in triplicate on the plate, and the experiment was repeated three times. All the fold changes were relative to the NBL stage. ^*^Data are significantly different from NBL (*P* < 0.05).

As shown in Figure 5, the Ts-CLP was expressed in every developmental stage, and the expression level peaked in L6h. Transcription reached a minimum level in NBL that was much lower than the minimum levels detected in the other stages (*P* < 0.05, Figure 5). From L24h to Ad3 a slight increase was observed.

### Serum antibody levels in pigs infected with *T. spiralis* by ELISA with rTs-CLP

**Figure 6:**
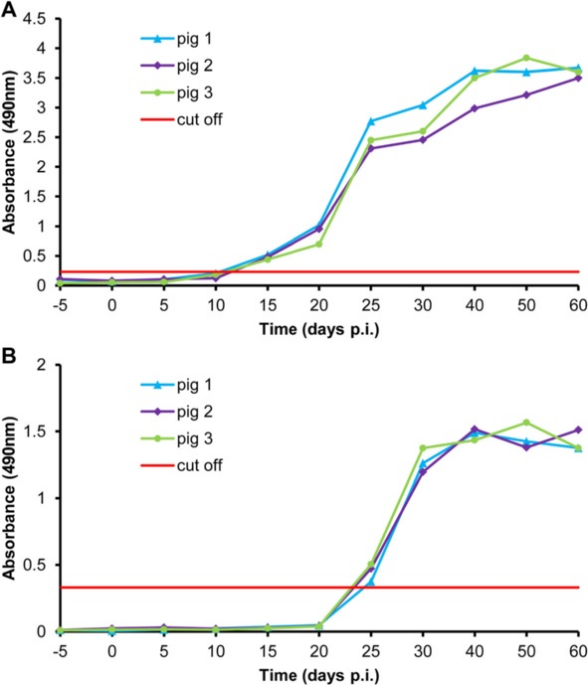
**Serum antibody levels in pigs infected with** ***T. spiralis*** **by ELISA with rTs-CLP.** Pigs were infected per os with 20000 ML of *T. spiralis*, and sera were collected prior to infection and at 5, 10, 15, 20, 25, 30, 40, 50 and 60 days p.i. Serum samples obtained prior to infection were used as negative controls. rTs-CLP **(A)** or ES **(B)** was used as antigen.

The results in Figure 6 represent the antibodies kinetics in pigs that were experimentally infected with *T. spiralis*, using rTs-CLP and the ES products of ML. The ELISA results demonstrated an early humoral response to rTs-CLP. Seroconversion was detected as early as 15 days p.i. In contrast, *Trichinella* infection could not be detected until 25 days p.i. using the ES products of ML.

### Immunoprotection analysis

**Figure 7:**
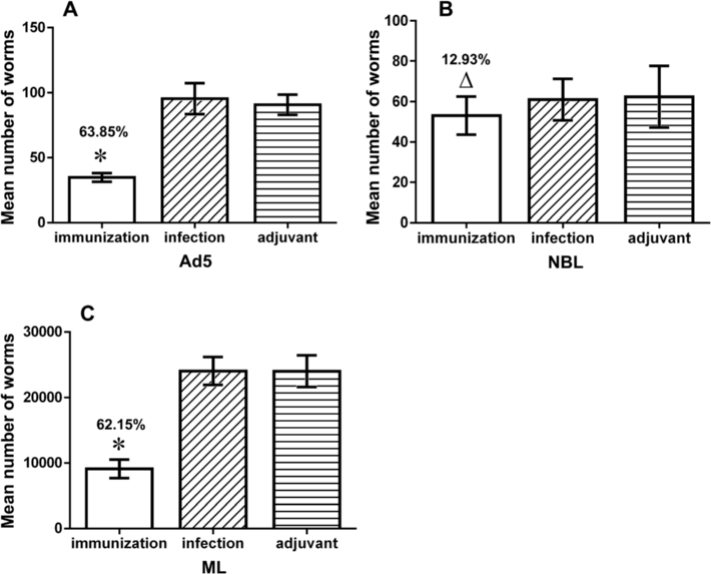
**Worm burden in immunised mice after T. spiralis challenge.** BALB/c mice were immunized with rTs-CLP. Reduction rates of Ad5 **(A)**, NBL **(B)**, and ML **(C)** are shown and were calculated relative to PBS control group, respectively. ^*^Data are significantly different from the PBS control group and adjuvant control group (*P* < 0.05). ^△^Data are not significantly different from the PBS control group and adjuvant control group (*P* > 0.05). Data for each adjuvant control is not significantly different from the infection control (*P* > 0.05).

The protective response induced by rTs-CLP against *T. spiralis* infection was investigated in BALB/c mice. The recovery of ML and Ad5 and the fecundity of females (NBL/female) were evaluated. The results showed that the reduction rates of both Ad5 (64.28%, Figure 7A) and ML (61.21%, Figure 7C) in the immunisation groups were significantly different from those in the PBS control group and adjuvant control group (*P* < 0.05). In contrast, the reduction rate in NBL/females was 16.46% (Figure 7B). There were no significant differences in each parameter between adjuvant control group and PBS control group (*P* > 0.05).

## Discussion

After being released in the host stomach, *T. spiralis* ML migrate to the small intestine and enter intestinal columnar epithelial cells. This represents the earliest interaction between the parasite and host, and therefore, it is important to identify the antigens expressed during this stage. To demarcate this stage of infection, we counted the number of intestinal infective larvae at different time points. We found that the majority of the worms had invaded the intestinal columnar epithelial cells at 6 h p.i. and that the number of the worms did not increase after 6 h p.i. As a result, we considered that L6h represent the early stage of invasion, which contain the larvae, invade the intestine in 0–6 hours. The determination of this stage is crucial for the identification of antigens important for early diagnosis.

In total, 33 positive clones were selected from the L6h cDNA library, and 20 of 33 clones encode the same 46-kDa protein which was termed Ts-CLP.

The assessment of *Ts-clp* transcription by real-time quantitative PCR demonstrated that it was transcribed during the entire life cycle of *T. spiralis*, even though the transcription level in NBL was significantly lower than that at other stages (*P* < 0.05, Figure 5). This expression profile was also demonstrated by the EST data. A Search of the expressed sequence tag (EST) databases of *T. spiralis* in GenBank (Mitreva *et al*. [26] and Park *et al*. [27]) revealed 73, 57 and 0 ESTs of this gene in the ML (5 020 ESTs), Ad (2 981 ESTs) and NBL (3 234 ESTs) databases.

The expression levels demonstrated by the intensity of the immunofluorescence signals of Ts-CLP in slides were consistent with real-time PCR results and EST databases. The confocal microscopy results clearly showed that native Ts-CLP localised to the stichosome (Figure 4C, 4G). According to the images showing the morphology (www.*Trichinella*.org) and the results reported by Guiliano [28], the localisation was in β-stichocytes of the stichosome. We failed to detect secreted Ts-CLP protein in NBL (Figure 4D), the capsule and muscle tissue (Figure 4F-H), but we observed Ts-CLP signals in the intestinal epithelium (Figure 4B). This finding suggested that Ts-CLP might be expressed and stored in the stichosome but is not secreted during the muscle phase. The low level of transcription and translation of *Ts-clp* during the NBL stage was probably due to the fact that the stichosome was not fully developed. The stichosome is either absent [14] or relatively weakly developed in NBL, and it becomes well-developed 4–5 days post-parturition and is maintained in ML and Ad [29]. As a result, we propose that Ts-CLP is synthesised in the β-stichocytes.

Interestingly, in our previous immunoscreening study, *Ts-clp* cDNA was selected from the *T. spiralis* Ad3 cDNA libraries only at a low frequency (7 of 32 positive clones), and it was not detected in libraries of NBL and ML [24]. Therefore, Ts-CLP was considered to be secreted only during intestinal phase and to play a certain role in invasion and/or parasitism of the small intestine.

We detected the antigenicity of rTs-CLP and examined immune protection with rTs-CLP in a mouse model. The rTs-CLP could be recognised by sera from the hosts infected with *T. spiralis* (Figure 3A), and the anti-rTs-CLP serum could recognise a ~46-kDa protein from the ES products of Ad5, ML and L6h (Figure 3B). Immunisation with rTs-CLP partially prevented the migration and/or penetration of *T. spiralis* into the intestinal epithelium and muscle cells. After the challenge infection with *T. spiralis* infective larvae, the mice immunised with rTs-CLP protein displayed a 64.28% reduction in Ad5 worm burden and a 61.21% reduction in ML worm burden, whereas there was no obvious correlation between the function of rTs-CLP and the fecundity of female worms (Figure 7). The ML reduction observed in present study is higher than that previously reported [30-32]. These values might be influenced by the isolates of the nematode employed, the inoculum dose of antigen and the dose of worms used for the challenge infection, among others.

The IgG levels of infected pig sera against rTs-CLP indicated that rTs-CLP could be considered as a good diagnostic antigen, especially during early stages of infection. The rTs-CLP could be detected as early as 15 days p.i. (Figure 6A), but the ES products of ML could only be detected after 25 days p.i. (Figure 6B). These results were similar to those reports showing that ES antigens of ML could be detected only after 3–4 weeks [8,9]. Therefore, it is exciting that it is an ideal recombinant antigen yet reported for the early detection of *T. spiralis* infection.

Tyvelose has been identified as a carbohydrate epitope of ES antigens [33,34], while rTs-CLP expressed in *E.coli* lacks glycosylation. Although the prokaryotic system resulted in the loss of carbohydrate epitopes and its sensitivity, recombinant proteins still possess the major epitopes and specificity. One of the strategies to protect the consumers against *Trichinella* infection is the identification of antigens that are efficient and convenient for serological diagnosis in the slaughterhouse, among which early diagnosis is fundamental [35]. However, the use of serological tests to detect *Trichinella* infection in domestic and wild animals and in humans has not yet been standardised [36]. The rTs-CLP has potential in this regard, it is easier and cheaper to prepare, compared to ES antigens. Thus, rTs-CLP could be considered as an available candidate for early diagnosis that fills the ‘diagnostic window’ and can even be used for detection during the entire life cycle.

Interestingly, a multi-cystatin-like domain protein (MCD-1, GenBank: ABH07907.1) was identified from ES products of *T. spiralis* ML in a proteomics analysis, but no information about application of MCD-1 in diagnosis was mentioned [37,38].

Ts-CLP is a novel cystatin-like protein that contains three repeated amino acid domains (Figure 2A). Ts-CLP have two motifs (GXA/TXYXD) that are located around each domain linker (Figure 2B), similar to the GXNXFXD motif required for the pH-dependent auto-processing of secreted cathepsins [39]. MCD-1 undergoes pH-dependent auto-processing under different pH conditions, but complete auto-processing can only occur under highly specific conditions *in vivo* [38]. As shown in Figure 3B, some low-molecular-weight bands of ES products of worms were also recognised by rabbit anti-rTs-CLP antibody. Whether this process occurs in Ts-CLP should be verified.

In addition, many parasitic cystatins can induce production of the Th2 cytokine IL-10 [40]. IL-10 can inhibit TNF-α production, which is a key feature of the pathogenesis of IBD [41,42], and therefore parasitic cystatins may be good candidates for IBD therapy. The same up-regulation of IL-10 was observed in *T. spiralis*-infected mice [43]; however, whether it was caused by Ts-CLP remains to be confirmed.

To date, only one cystatin-like protein of *T. spiralis* have been reported, while none cystatin-like protein has been reported for the un-encapsulated species *Trichinella pseudospiralis*. It is important to note that strong inflammatory responses result from infection with *T. spiralis* but not from infection with *T. pseudospiralis* [44,45,43,46,47]. Unexpectedly, when investigating the ESTs of *T. pseudospiralis*, we successfully discovered Ts-CLP from NBL with 12 ESTs (total of 17 330, deposited by Liu *et al*.); 31 ESTs from Ad3 (total of 20 046 ESTs, unpublished data), and 27 ESTs from ML (total of 19 994 ESTs, unpublished data). Therefore, the primary conclusion could be made that Ts-CLP produced during the life cycle of both *T. spiralis* and *T. pseudospiralis*.

## Conclusions

Ts-CLP is a highly reactive and abundant antigen recovered from the early stage of *T. spiralis* infection and represents a hopeful candidate for the diagnosis and prevention of *T. spiralis* infections.
